# Meristem maintenance, auxin, jasmonic and abscisic acid pathways as a mechanism for phenotypic plasticity in *Antirrhinum majus*

**DOI:** 10.1038/srep19807

**Published:** 2016-01-25

**Authors:** Julia Weiss, Raquel Alcantud-Rodriguez, Tugba Toksöz, Marcos Egea-Cortines

**Affiliations:** 1 Genetica Molecular, Instituto de Biotecnología Vegetal, Universidad Politécnica de Cartagena, 30202 Cartagena, Spain

## Abstract

Plants grow under climatic changing conditions that cause modifications in vegetative and reproductive development. The degree of changes in organ development i.e. its phenotypic plasticity seems to be determined by the organ identity and the type of environmental cue. We used intraspecific competition and found that *Antirrhinum majus* behaves as a decoupled species for lateral organ size and number. Crowding causes decreases in leaf size and increased leaf number whereas floral size is robust and floral number is reduced. Genes involved in shoot apical meristem maintenance like *ROA* and *HIRZ,* cell cycle (*CYCD3a*; *CYCD3b, HISTONE H4*) or organ polarity (*GRAM*) were not significantly downregulated under crowding conditions. A transcriptomic analysis of inflorescence meristems showed Gene Ontology enriched pathways upregulated including Jasmonic and Abscisic acid synthesis and or signalling. Genes involved in auxin synthesis such as *AmTAR2* and signalling *AmANT* were not affected by crowding. In contrast, *AmJAZ1, AmMYB21, AmOPCL1* and *AmABA2* were significantly upregulated. Our work provides a mechanistic working hypothesis where a robust SAM and stable auxin signalling enables a homogeneous floral size while changes in JA and ABA signalling maybe responsible for the decreased leaf size and floral number.

In contrast to animals, plants produce organs throughout development and environmental conditions play a key role in the size and type of organs produced. The degree of developmental plasticity of lateral organs seems to be determined by a combination of organ identity, the species under study and the type of environmental conditions that may affect its ontogeny. Some organs are highly robust and show little variation. One proposed mechanism to establish robust traits is the existence of genetic redundancy and highly interactive genetic networks^1^. The other side of the coin is plasticity. Many developmental processes are highly plastic^2^ such as root formation, leaf development or flowering time as they respond to environmental cues.

Aerial organs such as leaves and flowers are generated from the shoot apical meristem (SAM). Plants may show adaptation to changing environments in the SAM via modifying the output of organ number and/or size. Cells produced in the SAM, are displaced to side positions and become recruited to form lateral organ primordia. In *Antirrhinum majus*, stem cells are maintained undifferentiated by the homeobox gene *ROSULATA (ROA)*^3^, expressed in the quiescent zone, just underneath the shoot apical meristem. Orthologs of *ROA* like *WUSCHEL* from Arabidopsis or *TERMINATOR* from Petunia show a conserved function^4^,^5^. Cells in the SAM retain a meristematic identity due to the expression of *HIRZINA (HIRZ)* and *INVAGINATA (INA)*^6^, two homeobox genes belonging to the *KNOTTED,* and *SHOOT MERISTEMLESS* family. The identity of lateral primordia will depend on the developmental stage of the SAM. If the floral program is initiated, lateral primordia will adopt a floral identity.

The development of lateral organs seems to be initiated by an increase in the local levels of auxins^7^. Local changes in auxin synthesis maybe important in adaptation to the environment as the Arabidopsis auxin synthesis gene *TRYPTOPHAN AMINOTRANSFERASE 1 TAA1* plays a role in shade avoidance^8^. The *AP2* transcription factor *AINTEGUMENTA* has a dual function in activation of the polarity genes and the establishment of floral organ identity^9^. It also plays a role in lateral organ size via control of cell division and expansion in Arabidopsis, Petunia and *Antirrhinum*^10^,^11^,^12^, and is downstream of the auxin signalling pathway^13^. As lateral organs initiate, organ polarity genes take over and a combination of transcription factors and siRNAs establish the growth planes of lateral organs^14^. The *YABBY* genes fulfil a key role in establishing the proximo-distal polarity of lateral organ formation and in *Antirrhinum* the gene *GRAMINIFOLIA* plays a function in lateral organ formation^15^.

Floral organs are formed as a result of the coordinated expression of several genes that give rise to sepals, petals, stamens and carpels whose identity is established by combinations of MADS-box genes^16^. The proper activation of MADS-box genes are also responsible for the final size and shape of the floral organs, and play a key role in maintenance of basic cell division and expansion in the flower^17^,^18^.

Although the basic functioning of meristem maintenance is understood, the influence of environmental cues on meristem output, understanding it as a combination of lateral organ formation and organs that attain a certain size, is not. Both biotic and abiotic stresses tend to cause a decrease in the number and/or size of lateral organs produced by plants. Integration of stress is thought to occur via changes in the levels of plant growth regulators like brassinosteroids, jasmonic acid (JA), abscisic acid (ABA) or auxins, all of which can show interactions with each other^19^,^20^. Thus changes in signalling may account for modified SAM activity.

We found by serendipity that *Antirrhinum majus* flower size was robust and highly resilient to changes under different growth conditions, whereas vegetative development, specifically leaf size was strongly affected by the environmental conditions. We established a system based on plant crowding, used in ecology and in agriculture to test its effects on floral number and size. Analysis of gene expression showed little or no changes in genes involved in meristem maintenance, cell division and auxin signalling suggesting that floral organ size robustness depends on meristem homeostasis. A general transcriptomic analysis showed enrichment and overexpression of genes involved in jasmonic and abscisic acid signalling and synthesis. These changes may be responsible for the decreased leaf size and floral numbers that occurred as adaptation to intraspecific competition.

## Results

### Effect of crowding on growth and development

We had previously found that intense leaf removal has little or no effect on floral size in *Antirrhinum majus*^21^. In a large set of F2 populations used to create an *Antirrhinum* map^22^, we found that plants left on small pots after picking to larger trays where segregations took place ended up with a very small vegetative size, but floral size appeared to be normal. Experiments were carried out several times in autumn, winter and spring in order to estimate effects of crowding on development and to verify the preliminary observations. We used pots with 1, 5 or 10 plants but we performed all analysis on control (1 plant per pot) and the most extreme treatment (10 plants per pot).

**Figure 1:**
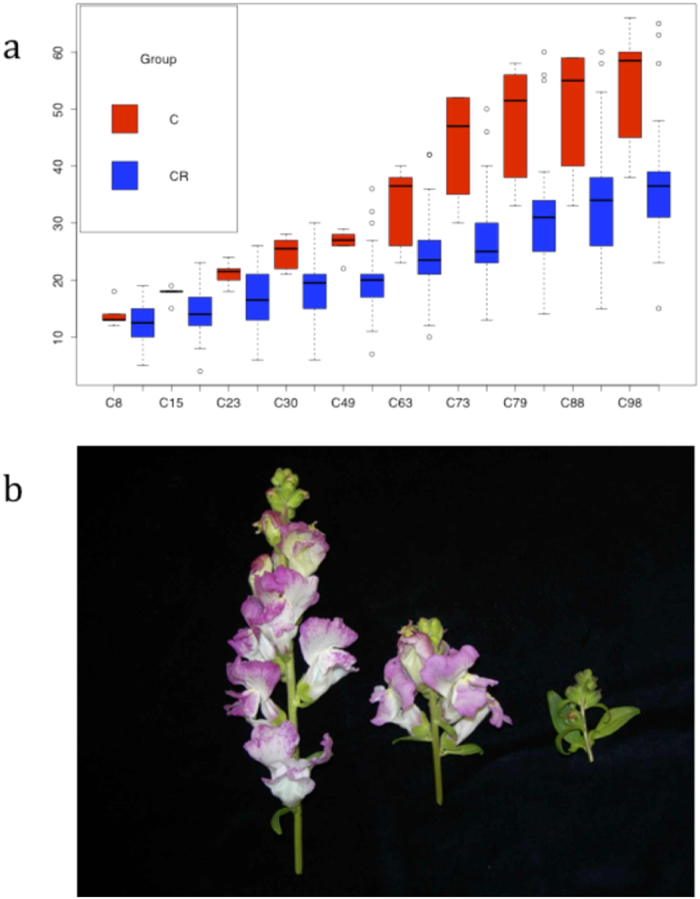
(**a**) Growth kinetics of C, control and CR, crowded plants, numbers refer to days after sowing. The Y axis refers to height in cm. (**b**) Typical inflorescences of crowded plants at 1, 5 and 10 plants per pot. The picture shown in figure B was taken by MEC.

Growth of plants subject to crowding was analysed during development (Table 1). We measured the length of the first three internodes and found that they were significantly shorter (Table 1), indicating that the effect of crowding on growth occurred from early stages of development. A detailed kinetic analysis of shoot length showed that at early stages of growth (8 days), crowded plants were significantly shorter than controls (P = 0.0321). However, this trend was abrogated during later growth as from day 15 onward crowded plants caught up with the controls and differences were non significant (Fig. 1a).

Changes from double decussate to spiral phyllotaxis have been described in *Antirrhinum* as a point of floral transition^23^. We did not observe changes in the number of double decussate leaves indicating that transition to flower was not affected by the crowding conditions (Table 1). However we found a significant increase in the number of spiral leaves before flower primordia appeared (p < 0.05), pointing to a delayed acquisition of the floral program in the SAM. As a result the total number of leaves produced under crowding was significantly larger (p < 0.01). Leaf area was significantly decreased indicating that despite the increased number of leaves, there was a strong reduction in size.

We measured ten parameters describing floral size (Fig. 1b). Two floral parameters, sepal length and dorsal petal expansion were significantly larger in crowded plant (Table 2). Both stamen and gynoecium length were significantly shorter in crowded plants as compared to control flowers, albeit the actual differences were of 5.2% (P = 0.017) in stamens and 4.5% in gynoecia (P = 0.023). The number of flowers per plant was strongly reduced to 52% in crowding conditions (P = 0.001) (Table 1). To assess a cause-effect relation between foliar area variations and flower production we made a correlation analysis between both characters. Plants grown under normal conditions did not show any type of correlation between floral number and leaf area (Spearman Coefficient 0.0629, p = 0.739) but under crowding conditions we found a statistically significant positive correlation (p = 0.013). The low value of the Spearman coefficient (0.5853) suggests that there are other factors affecting floral number production under crowding beyond leaf area reduction.

### Effect of environmental stress on basic meristem maintenance

**Figure 2:**
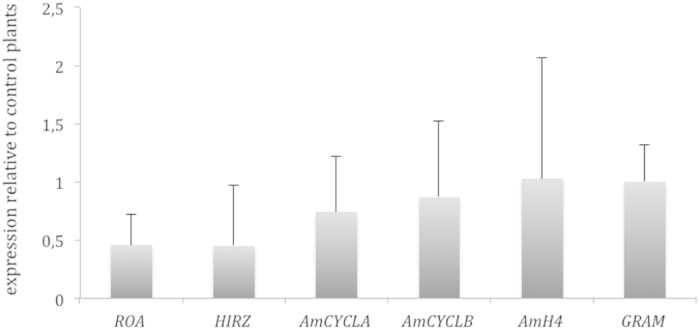
Quantitative expression analysis of genes involved in meristem maintenance, cell division and organ polarity in inflorescence meristems under crowding conditions. The level of 1 corresponds to expression levels in the control plants. Error bars correspond to standard deviation.

We analysed the effect of crowding on gene expression in inflorescence meristems harvested when floral primordia started to appear. We measured gene expression levels that define basic meristem functions, meristem maintenance by *ROA* and meristem growth by *HIRZ*. We found that both *HIRZ* and *ROA* showed a trend towards down regulation in crowding but this trend was not significant (HIRZ P = 0.513; ROA P = 0.465) (Fig. 2). We measured three cell division markers, *CYCLIN D3b* (*CycD3b*) expressed in the SAM and lateral primordia (*CycD3b*), *CYCLIN D3a*^24^ and *HISTONE H4* (*H4*), marking mitotic index^25^. Both *CyCD3a* and *CyCD3b* showed non significant down regulation, indicating that local levels of cell division could be maintained in the centre of the SAM and lateral primordia. The expression of H4 was similar in control and crowded plants (relative expression level 1.549 P = 0.518) indicating that general mitotic index was not affected (Fig. 2).

Formation of lateral primordia is marked at very early stages by the *YABBY* gene *GRAMINIFOLIA*, involved in lateral organ polarity^15^. The expression levels of *GRAM* were unchanged (relative expression level 1.004 P = 0.459) indicating that the relative effect of crowding on lateral primordia initiation could not be identified by changes in levels of organ polarity acquisition.

Altogether we can conclude that crowding had non-significant effect on meristem maintenance genes, cell division or organ polarity establishment.

### Transcriptomic analysis of SAM under crowding conditions

We performed a large scale transcriptomic analysis to uncover possible mechanisms of stress integration in the SAM. A total of 1134 genes were significantly upregulated and 361 were down regulated (Supplementary Table S1). A gene ontology enrichment analysis identified biological processes in the up and down regulated genes (Supplementary Table S2). Despite showing a lower number of down regulated genes, the number of significantly different down regulated GO terms was substantially larger with 11 biological processes, 5 cellular components and one molecular process affected There were only 4 biological processes, 2 cellular components and one molecular function with significant up regulation.

**Figure 3:**
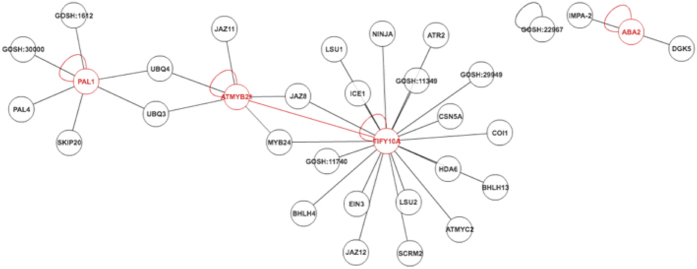
GO-based biological pathways with significant increase in gene expression in crowded plants. Red coloured are up-regulated genes in list that interact with others in the list. Black indicates those genes in the input that have an interaction but are not up-regulated.

We used the corresponding Arabidopsis orthologs to identify enriched biological pathways^26^. The result of the analysis showed two sets of genes significantly up regulated, but discrete downregulated pathways were not found. One upregulated pathway corresponds to genes involved in JA signalling including the MYB transcription factors *JAZ1* and *MYB21* as central genes. The second upregulated pathway was related to ABA synthesis and was centred on *ABA2* (Fig. 3). These results indicated that signalling into the SAM could occur via JA and ABA. Surprisingly auxin signalling or synthesis was apparently unaffected.

As we used multiple displacement amplification for cDNA amplification and biases have been found to occur as a result of this technique in GC rich templates^27^, we tested several genes that were not significantly modified and those that appeared as upregulated networks in the microarray.

### Auxin signalling is not modified by stress

**Figure 4:**
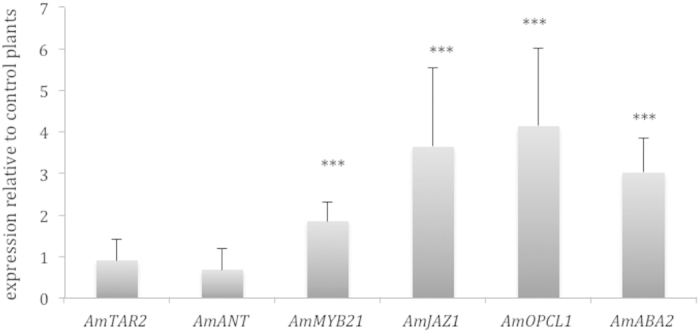
Quantitative expression analysis of genes involved in auxin, JA and ABA signalling and synthesis. The level of 1 corresponds to expression levels in the control plants. Stars indicate p < 0.001 (***).

There are several hormone-signalling pathways that play a role in meristem function. Amongst them auxin plays a key role in lateral organ initiation. Despite the importance of auxins in lateral organ formation and general plant development there was no obvious change in expression of genes involved in auxin signalling, transport or synthesis. We identified 16 genes with annotation related to auxins (Table 3). Only two genes showed significant changes in gene expression. *INDOLEACETIC ACID-INDUCED PROTEIN 16* was significantly down regulated (−2.04 fold) and *SMALL AUXIN UPREGULATED RNA 51* was significantly up-regulated (2.03 fold). Genes with well established roles in auxin transport like *PIN-FORMED 4* or signal transduction like *AUXIN RESISTANT 2* did not show significant differences in gene expression. Furthermore, two Arabidopsis genes, *AUXIN INDUCIBLE 2-11*, and *DORMANCY-ASSOCIATED PROTEIN 1* were apparently duplicated in *Antirrhinum*. Whilst one paralog was non significantly up regulated (Snap112107_cn5436 and Snap112107_cn2840), a second one was non-significantly down regulated (Snap112107_cn5435 and Snap112107_cn2580) (Table 3) suggesting that altogether the auxin signalling pathway was not affected by crowding. In order to further verify this result, we identified an *Antirrhinum* clone (AJ794078) with high homology to the *TRYTOPHAN AMINOTRANSFERASE (TAA)* and *TRYTOPHAN AMINOTRANSFERASE RELATED* genes involved in auxin synthesis and shade avoidance^8^,^28^. The expression of AJ794078 was not significantly affected in the microarray (−1.19 fold downregulation). We performed a phylogenetic analysis of AJ794078 to identify its degree of homology with the *TAA1-TAR* family from Arabidopsis. The *Antirrhinum* clone clearly clustered together with *TAR2* from Arabidopsis and in a different clade from *TAA1* (Supplementary Fig. 1). We confirmed the expression level of *AmTAR2* under crowding, which was virtually identical to control plants (expression 0.901; P = 0.498) indicating that auxin biosynthesis was not affected by crowding (Fig. 4). As auxin signalling maybe localized in primordia, we tested the expression of *AmAINTEGUMENTA*, an *AP2* transcription factor involved in auxin signaling and lateral organ formation^10^,^13^,^29^. As found for *AmTAR2* expression. *AmANT* was non-significantly down-regulated (expression 0.901; P = 0.546) indicating that auxin synthesis and signalling was not affected by the imposed stresses.

### Crowding activates JA and ABA signalling

**Figure 5:**
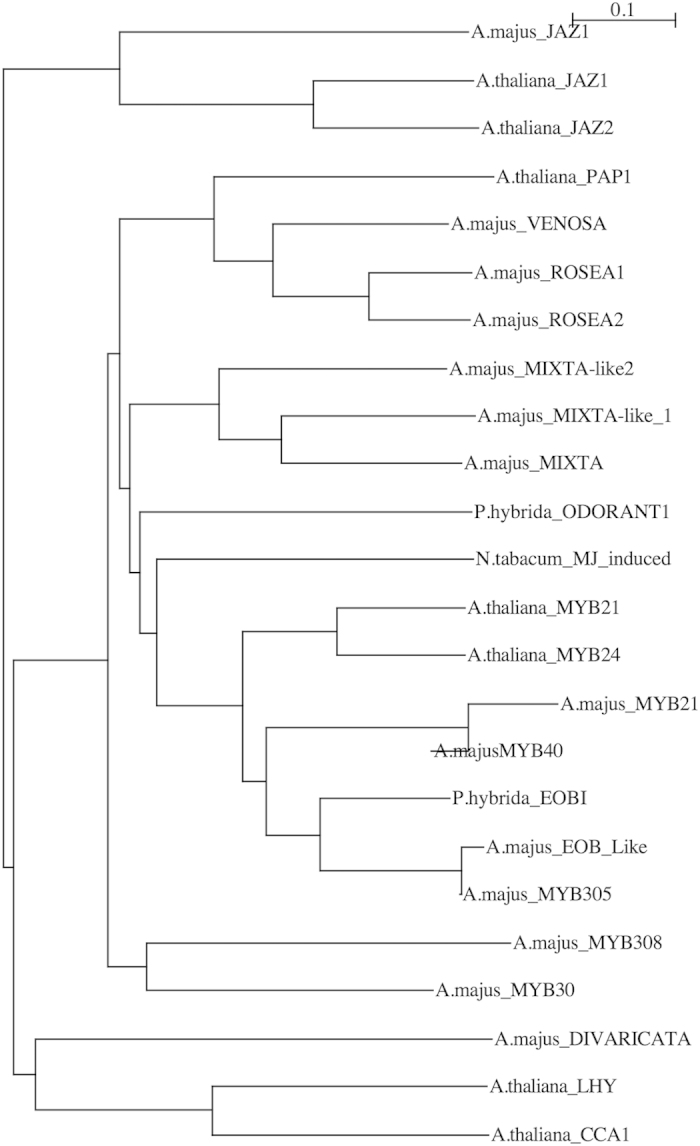
Phylogenetic analysis of AmJAZ1 and AmMYB21.

The *Antirrinum* genes corresponding to the Arabidopsis *MYB* genes *JAZ1* (AJ787051), *MYB21* (AJ797639) and the short chain alcohol dehydrogenase *ABA2*/*GIN1* (AJ802690) were used to verify the degree of sequence homology with the Arabidopsis genes by phylogenetic analysis. The EST corresponding to *AmJAZ1* was found to be on a single clade together with *AtJAZ1* and *AtJAZ2* (Fig. 5). The putative *AmMYB21* was in a clade together with *AtMYB21* and *AtMYB24* from Arabidopsis and was clearly separated from other *MYB* genes like *VENOSA* or *ROSEA* involved in anthocyanin patterning and colour intensity in petals^30^.

A quantitative PCR analysis showed significant up-regulation of both genes that closely corresponded to those found in the microarray analysis (Fig. 4). Indeed *AmMYB21* showed significant overexpression in the microarray of 3.43 fold and of 1.84 by qPCR with a p value of 0.000, *AmJAZ1* had a 2.81 up regulation in the microarray and a 3.63 upregulation in the QPCR analysis (p = 0.001).

There are several 4-coumarate C0 ligases in the Arabidopsis and the *OPC-8:0 COA LIGASE1* (*OPCL1*) is involved in jasmonic acid biosynthesis^31^,^32^. We identified an *Antirrhinum* gene that showed high homology with the *OPCL1* gene from Arabidopsis. We confirmed this finding by a phylogenetic analysis that showed a high homology to *OPCL1* and a clear separation of other clades containing other acyl CoA ligases that are not involved in JA synthesis (Supplementary Fig. 2). An expression analysis showed that it was significantly up-regulated in crowded plants as compared to control plants (4.13 fold P = 0.001).

The *ABA2/GIN1* gene encodes a short-chain alcohol dehydrogenase catalyzing the conversion of xanthoxin to abscisic aldehyde^33^,^34^. We found two paralogs in *Antirrhinum* (Fig. 4). One of them (*A.majusABA2*; AJ802690) was significantly upregulated in the microarray analysis (3.13 fold). The corresponding gene was found to be 3.00 fold up regulated (p = 0.001) in QPCR assays.

Our results show first that the multiple displacement amplification may be suitable for amplification of cDNA libraries. And second, both JA synthesis, signalling and ABA synthesis are upregulated in crowded plants indicating that the identified decrease in leaf area and floral number may be the result of adjustments governed by these two pathways. A robust auxin signalling and meristem maintenance may be required to acquire normal floral size.

## Discussion

There are several paradigms in plant biology regarding growth and development. One is the role of the SAM as the generator of cells forming lateral organ primordia. Second is the fact that many species display correlations between vegetative and reproductive organ phenotypes^35^,^36^. And third is that the so-called floral organ identity genes may impose a morphogenetic program that is thought to be a departure from a default vegetative pathway^18^. Flowers and lateral organs can be considered as different phenotypic modules from a developmental and evolutionary perspective provided they have uncoupled mechanisms of control. In the present work we have identified the potential of *Antirrhinum majus* as a model to study decoupling of vegetative and reproductive development. The current hypothesis is that floral size may play a key role in pollination, and changes may modify fitness. Although the number of flowers is not related to leaf area under normal growth conditions our results clearly show that crowding imposes growth constrains where floral number becomes somehow dependent on leaf area^21^. This also indicates that testing coupling or decoupling requires several growth conditions whereby covariations may appear as a result of limiting resources.

Although our initial hypothesis was that changes in organ number production would be reflected by changes in basic meristem functions and cell division, our data proved us wrong. Our current interpretation is that the complete SAM has a coordinated reduction in functions in such a way that it maintains a coherent relationship between stem cell formation, lateral recruitment, cell division and primordia initiation. Furthermore cell division is carefully controlled in all different areas and does not show changes overall. If meristem maintenance and cell division are maintained it may explain the formation of perfect flowers irrespective of growth conditions.

The signalling and synthesis of several plant hormones is involved in meristem function, including auxins, brassinosteroids or JA. Light governs stem cell division via local changes in auxin levels involved in leaf positioning and phylotaxis^37^. Thus we would have expected changes in auxin synthesis or signalling. However neither the transcription level of *AmTAR*, an aminotransferase-related gene involved in auxin synthesis, nor *AmANT*, a gene responsive to auxin signalling and organ primordia initiation, were altered under the two treatment conditions.

Taken together our results indicate that a strong homeostasis of SAM maintenance and auxin signalling may be a requirement for floral size maintenance. The fact that leaf number is not decreasing whereas floral number does, indicates that there are two distinct mechanisms for the control of lateral primordia that may be dependent on the floral identity program. The vegetative phase may be dependent on a certain number of lateral organs that should be achieved in order to start flowering. Flowering time mutations and environmental conditions affect the number of leaves produced in Arabidopsis, *Antirrhinum* and other plants. The floral identity program may have an intrinsic mechanism controlling the number of cells per primordium thus ensuring achievement of the correct floral size.

JA signalling plays a dual role as they inhibit growth by suppressing mitosis in apical meristems^38^, but is also required for stamen development^39^,^40^. We found a significant up regulation of *AmJAZ1, AmMYB21* and *AmMYB24* involved in Jasmonic acid signalling at various levels and of *AmOPCL1* involved in early steps of JA synthesis. JAZ proteins are repressors of JA signalling^41^,^42^. But JAZ proteins interact with AtMYB21 and AtMYB24 controlling anther development^43^. The phylogenetic relationship of *A.majusMYB21* with *P.hybridaEOBI, A.majusMYB305* or *Phybrida_ODORANT1* is close enough to suggest additional functions of *A.majusMYB21*. In fact *AtMYB21* is required for terpenoid synthesis in Arabidopsis^44^.

Altogether crowding may be considered a combined stress and the emergence of the ABA synthesis pathway as upregulated in crowded plants is probably expected. ABA is a major integrator of abiotic stress in plants^45^. The *ABA2* gene is involved in ABA synthesis^33^,^34^ and plays an additional role in sugar sensing^46^,^47^. Activation of the ABA synthesis pathway should be responsible for improved resilience to abiotic stress. This translates in *Antirrhinum* plants with smaller but not fewer leaves. It may also define the pace of lateral organ formation once flowering starts, causing a general decrease in the number of flowers produced that are otherwise perfect. Pending further experimental evidence based on gain and loss of function of genes involved in the pathways analysed, we can speculate that a combination of robust behaviour and plastic expression of auxins, JA, ABA and meristematic functions may be involved in robustness and phenotypic plasticity of plant aerial organs.

## Materials and Methods

### Plant material, growth conditions and treatments

Seeds of *Antirrhinum majus* inbred line 165E were germinated on fine vermiculite and transplanted after two weeks to the final growth conditions on pots. Plants were watered as required with an automatic drip irrigation system in a greenhouse.

We transplanted 40 pots (650 ml volume) with one plant, and 20 with five or ten plants when the first pair of true leaves had emerged. The different vegetative parameters were measured in 30 individuals when plants flowered and at least one flower on the primary stem had opened. Twenty three percent of the plants in the 10 plants/pot treatment developed full leaf number but did not flower and were included in the measurements of plant size.

### Plant measurements

Total height was measured from the base to the top of the plants. Growth kinetics was recorded by measuring plant height every 7–14 days. Once the inflorescence meristems formed we measured additional vegetative parameters. Leaves were counted including leaves in the double decussated vegetative part and spirally organized single leaves in the inflorescence. Bracts subtending flower were not counted as leaves.

The leaves and bracts of the plants were cut as close to the stem as possible and all together were ordered on a paper sheet using a ruler as an internal standard. Leaves were scanned and leaf area was analyzed using the ImageJ program (http://rsb.info.nih.gov/ij/).

Once floral organs were fully developed^48^, the size of the different organs and parts of the flower were measured with a millimetre calliper as described^29^. The parameters measured were: 1, tube length; 2, ventral petal length; 3, petal height; 4, sepal length; 5, tube width; 6, dorsal petal length; 7, ventral petal expansion; 8, dorsal petal expansion; 9, stamen length and 10, gynoecium length.

### Statistical analysis

We performed statistical analysis with the R program (www.r-project.org), comparing 10 plants/pot against 1 plant/pot. One-way ANOVA was used for all the parameters that showed a normal distribution, and the ‘Kruskall and Wallis analysis’ in case that one of the parameters under comparison deviated from a normal distribution. The parameters were considered significantly different among treatments when the P value was smaller than 0.05.

### Microarray analysis

Inflorescence shoot apical meristems of *Antirrhinum* plants were sampled when the first flower opened, total RNA was extracted and first strand cDNA was synthesized as described previously. We gathered enough apical meristems to obtain 60–100 mgr of tissue. The number of meristems required was about 50. As the quantity of mRNA obtained and cDNA was minimal (0.1–1 ngr), we used Multiple Displacement Amplification to obtain enough cDNA for microarray analysis as described previously^49^. Microarray analysis was performed on two control samples and three crowded samples by MoGene using a microarray based on 12,497 Antirrhinum genes publicly available, and comprising 11959 single genes from *A.majus* (Omnibus accession number GSE36356)^50^,^51^. Microarray data were deposited in the NCBI Gene Expression Omnibus database (Accession number GSE72818).

### Bioinformatic analysis

Genes identified in *Antirrhinum* were compared by TBLASTN against Arabidopsis using translated sequences. In order to ascertain the degree of homology and the putative orthology of the sequences used for Q-PCR, we downloaded sequences obtained by TBLASTN from NCBI. Protein sequences were aligned using CLUSTALX, and phylogenetic trees were built using the built in Neighbour Joining algorithm^52^. Trees were rendered with NJPlot^53^.

Genes with significant differences in gene expression were blasted using BLAST2GO^54^, and the corresponding annotated genes were used to create a set of conserved Arabidopsis orthologs. The Arabidopsis orthologs were used to perform a Gene Ontology enrichment analysis and identify pathways defined in Gene Ontology with significant changes in gene expression using g:Profiler^26^.

### Quantitative PCR

Genes were amplified in a Stratagene Mx3000 qPCR machine (www.agilent.com), with sequence-specific primers (Table S1) synthesized by Invitrogen (www.invitrogen.com) using Takara SYBR-Green (www.thermofishcer.com). We used the gene ubiquitin as a control for normalization^29^. PCR efficiency was calculated as described before^55^. Statistical analysis of gene expression was performed using group-wise comparison with the REST program^56^. We performed two independent experiments with three biological and two technical replicas.

## Additional Information

**How to cite this article**: Weiss, J. *et al*. Meristem maintenance, auxin, jasmonic and abscisic acid pathways as a mechanism for phenotypic plasticity in *Antirrhinum majus. Sci. Rep.* **6**, 19807; doi: 10.1038/srep19807 (2016).

## Supplementary Material

Supplementary Fig 1 and Fig2

Supplementary Table S2

Supplementary Table S1

## Figures and Tables

**Table 1:** Effect of crowding on vegetative growth in Antirrhinum majus. Numbers on the first column correspond to 1 plant per pot 10 plants per pot and the difference.

	Internode 1	Internode 2	Internode 3	Total height	Number of flowers	Double decussate leaves	Spiral leaves	Total leaves	Leaf area (cm^2^)
1	1.54 ± 0.2	2.35 ± 0.38	2.99 ± 0.38	479. ± 5	16.97 ± 3.3	7.20 ± 1.27	5.63 ± 3.66	12.83 ± 2.56	145.70 ± 25
10	1.35 ± 0.2	2.0 ± 0.2	2.72 ± 0.57	295 ± 5	8.13 ± 4.71	6.96 ± 1.49	8.30 ± 3.44	15.26 ± 2.42	105.18 ± 28
1 vs 10	−12.18**	−14.62***	−8.89***	−38.33***	−52.08***	−3.38	47.41*	18.92**	−27.81***

Values represent average ± standard deviation. Differences expressed as treament versus control 100%. We performed student T-tests. P values correspond to *P < 0.05. **P < 0.01. ***P < 0.001. Measurements correspond to 30 individuals per treatment.

**Table 2:** Comparison of floral parameters between control and crowded grown plants. Values represent millimeters.

Organ	Control	Crowding	p-value
Tube length	14.8	15.2	0.706
Ventral petal length	25.9	27.3	0.774
Petal height	22.6	20.5	0.115
Sepal length	7.0	7.5	0.044
Tube width	12.1	11.7	0.175
Dorsal petal length	37.3	36.5	0.117
Ventral petal expansion	23.3	23.4	0.963
Dorsal petal expansion	26.9	29.6	0.012
Stamen length	23.5	22.3	0.017
Gynoecium length	20.2	19.3	0.023

**Table 3:** Auxin-related genes in Antirrhinum and changes in expression pattern under crowding.

Antirrhinum EST	Arabidopsis best hit and BLAST e value	Gene product	Regulation
Snap112107_cn1241	At3g04730 9e-70	INDOLEACETIC ACID-INDUCED PROTEIN 16	−2.04*
Snap112107_cn2384	At5g20630 1e-70	GERMIN 3	−1.56
Snap112107_cn5435	At5g43700 4e-48	AUXIN INDUCIBLE 2–11	−1.46
Snap112107_cn2580	At1g28330 8e-30	DORMANCY-ASSOCIATED PROTEIN 1	−1.26
Snap112107_cn1049	At5g65470 1e-111		−1.11
Snap112107_cn5183	At1g54070 2e-04	Dormancy/auxin associated family protein	1.07
Snap112107_cn2029	At1g77690 1e-133	LIKE AUX1 3	1.26
Snap112107_cn5444	At2g01420 1e-103	PIN-FORMED 4	1.26
Snap112107_cn3718	At1g04240 6e-58	INDOLE-3-ACETIC ACID INDUCIBLE 3	1.34
Snap112107_cn0753	At1g76520 1e-52	PIN-LIKES 3	1.41
Snap112107_cn1200	At3g23050 1e-85	AUXIN RESISTANT 2	1.46
Snap112107_cn5436	At5g43700 9e-54	AUXIN INDUCIBLE 2–11	1.47
Snap112107_cn5633	At3g09270 4e-36	GLUTATHIONE S-TRANSFERASE TAU 8	1.54
Snap112107_cn2840	At1g28330 2e-28	DORMANCY-ASSOCIATED PROTEIN 1	1.57
Snap112107_cn4733	At1g72420 1e-70	NADH:ubiquinone oxidoreductase intermediate-associated protein 30	1.77
Snap112107_cn1577	At1g75580 3e-37	SMALL AUXIN UPREGULATED RNA 51	2.03*

Regulation indicates negative or positive regulation and * indicates significant change in gene expression (P < 0.05).
