# Supplementary material for: Meristem maintenance, auxin, jasmonic and abscisic acid pathways as a mechanism for phenotypic plasticity in Antirrhinum majus
Source: Sci Rep. 2016 Jan 25;6:19807. doi: 10.1038/srep19807 (PMC4726321; doi:10.1038/srep19807)
Supplement: Supplementary Fig 1 and Fig2 [file srep19807-s1.doc]

Supplementary material

Meristem maintenance, auxin, jasmonic and abscisic acid pathways as a mechanism for phenotypic plasticity in *Antirrhinum majus*.

Julia Weiss, Raquel Alcantud-Rodriguez, Tugba Toksöz and Marcos Egea-Cortines

Figure S1. Phylogenetic Analysis of AmTAR2. The sequences used to build the tree correspond to:

Arabidopsis thaliana TAR2 AT4G24670; Rice_fishybones UniRef100_Q5VQG8; Zea_mays_Vanishingtassel2 TPA_exp gi|327478401; A.majus AJ794078-TAR2-like; Coffea_canephora gi|661883674; Vitis_vinifera_TAR2 gi|225456934; Citrus_sinensis gi|641850650; Ricinus communis gi|255540565; Citrus_clementina gi|567894804; A.thaliana_Alliin_lyase _gi|4220523; Capsella_rubella gi|565439663; Arabidopsis_thaliana_TAR2 _gi|18416401; Theobroma_cacao_TAR2 gi|590663932; A.lyrata_TAR2 gi|297799538; Solanum_tuberosum_TAR2 gi|565361383; Rosa_hybrida gi|684057902; Cicer_arietinum gi|502133776; Populus_trichocarpa gi|566197463; Physcomitrella_patens gi|168042587; Solanum_lycopersicum gi|460376389; A.thaliana_TAA1 AT1G70560; A.thaliana_TAR AT1G23320.1; Cicer_arietinum_TAR2 gi|502176528.

Figure S2. Phylogenetic analysis of A.majus OPCL1. The sequences used to build the tree correspond to:

S.moellendorffii 15419256; A.lyrata 16046259; A.lyrata2 16063847; C.papaya 16415162; R.communis 16822160; C.sativus 16973638; P.persica 17660246; M.guttatus 17675469; V.vinifera 17821255; M.esculenta 17966968; P.patens 18048481; C.sinensis 18091655; A.thaliana_OPCL1 AT1G20510.1; A.thaliana_4C_CoA_Ligase AT1G20480.1; A.thaliana_4C_CoA_ligase2 AT1G20500.1; A.thaliana_4C_CoA_ligase3 AT5G38120.1; T.halophila1 20185405T.halophila2 20185868; T.halophila3 20186633; T.halophila4 20186966; T.halophita5 20187298; T.halophita6 20187839; C.clementina 20789127; C.rubella 20886687; C.rubella2 20890164 ; C.rubella3 20892260; C.rubella4 20892277; C.rubella5 20892502; A.coerulea 22048581; M.domestica 22635871; M.domestica2 22660672; B.rapa_Chifu-401 22697242; B.rapa2 22700495; B.rapa3 22700992; B.rapa4 22722203; B.rapa5 22723148; B.rapa6 22724018; M.truncatula 23037985; S.tuberosum PGSC0003DMP400051055| ; G.max 26284573; G.raimondii 26793192; G.raimondii2 26806072; P.trichocarpa 27023535; P.trichoparpa2 27030501; P.vulgaris 27161910; S.lycopersicum 27308053; T.cacao 27457047;A.majus_AJ801733_2 ; Arabidopsis_OPCL1 AT1G20510
